# The Role of Complement in the Mechanism of Action of Therapeutic Anti-Cancer mAbs

**DOI:** 10.3390/antib9040058

**Published:** 2020-10-28

**Authors:** Josée Golay, Ronald P. Taylor

**Affiliations:** 1 Center of Cellular Therapy “G. Lanzani”, Division of Hematology, Azienda Socio Sanitaria Territoriale Papa Giovanni XXIII, 24127 Bergamo, Italy; 2 Fondazione per la Ricerca Ospedale Maggiore, 24127 Bergamo, Italy; 3 Department of Biochemistry and Molecular Genetics, University of Virginia School of Medicine, Charlottesville, VA 22908, USA

**Keywords:** therapeutic monoclonal antibodies (mAbs), complement, antibody dependent cellular cytotoxicity, phagocytosis, complement receptors

## Abstract

Unconjugated anti-cancer IgG1 monoclonal antibodies (mAbs) activate antibody-dependent cellular cytotoxicity (ADCC) by natural killer (NK) cells and antibody-dependent cellular phagocytosis (ADCP) by macrophages, and these activities are thought to be important mechanisms of action for many of these mAbs in vivo. Several mAbs also activate the classical complement pathway and promote complement-dependent cytotoxicity (CDC), although with very different levels of efficacy, depending on the mAb, the target antigen, and the tumor type. Recent studies have unraveled the various structural factors that define why some IgG1 mAbs are strong mediators of CDC, whereas others are not. The role of complement activation and membrane inhibitors expressed by tumor cells, most notably CD55 and CD59, has also been quite extensively studied, but how much these affect the resistance of tumors in vivo to IgG1 therapeutic mAbs still remains incompletely understood. Recent studies have demonstrated that complement activation has multiple effects beyond target cell lysis, affecting both innate and adaptive immunity mediated by soluble complement fragments, such as C3a and C5a, and by stimulating complement receptors expressed by immune cells, including NK cells, neutrophils, macrophages, T cells, and dendritic cells. Complement activation can enhance ADCC and ADCP and may contribute to the vaccine effect of mAbs. These different aspects of complement are also briefly reviewed in the specific context of FDA-approved therapeutic anti-cancer IgG1 mAbs.

## 1. Introduction

Human IgG1 monoclonal antibodies (mAbs), after antigen binding, have the ability to activate the classical pathway of the complement cascade and mediate complement-dependent cytotoxicity (CDC) [1,2]. They can also cross-link Fcγ receptors expressed by immune cells, including natural killer (NK) cells, monocytes/macrophages, and neutrophils, and thereby activate cell-mediated innate immunity [2,3,4]. IgG1 mAbs therefore have the ability to activate both the humoral and cellular immune system for the immunological control of tumor growth and metastasis. These two pathways may also interact with each other, with potential synergy [5]. This is why most therapeutic mAbs that target a tumor antigen have been designed to bear a functional or even enhanced human IgG1 Fc portion. The tumor-specific unconjugated mAbs approved by the Food and Drug Administration (FDA) and European Medicines Agency (EMA) are listed in Table 1. The first part of the table lists mAbs approved for hematological malignancies, and the second part lists mAbs approved to target solid tumors. The gold standard for these mAbs is the anti-CD20 rituximab, which was the first approved anti-cancer mAb (in 1997) and has shown considerable therapeutic activity in several B cell tumor subtypes, initially as monotherapy, and subsequently in combination with chemotherapy. Indeed multiple phase III clinical studies have demonstrated the clinical efficacy of rituximab in combination with chemotherapy or as maintenance therapy in B-cell non-Hodgkin’s lymphoma (B-NHL) (in particular follicular lymphoma and diffuse large B cell lymphoma) and, to a lesser extent, chronic lymphocytic leukemia (CLL), Burkitt’s lymphoma, and mantle cell lymphomas (reviewed in Past, Present, and Future of Rituximab-The World’s First Oncology Monoclonal Antibody Therapy [6]). Rituximab efficiently activates CDC, antibody-dependent cellular cytotoxicity (ADCC), and antibody-dependent cellular phagocytosis (ADCP) in vitro, and these different mechanisms must all contribute to its efficacy [4,7]. CD20 is well expressed in most mature B-cell leukemias and lymphomas and is nearly exclusively restricted to the B cell lineage, a factor likely contributing to the success of rituximab and other anti-CD20 antibodies.

Beyond their activation of innate immune mechanisms mentioned above, mAbs targeting tumor antigens may also act by blocking (neutralizing) the antigen receptor or enzymatic function through their Fab portion, either by interference with ligand binding or through internalization and degradation of the receptor or both, leading to inhibition of cell growth or of metastasis [8,9,10]. Some mAbs also induce direct cell death after antigen binding [11,12,13]. Table 1 reports the principal mechanisms of action that are thought to be at the base of the efficacy of the unconjugated IgG1 anti-tumor antigen mAbs approved for anti-tumor therapy. Some have antigen neutralizing functions, as expected, since they target important growth factor receptors such as human epidermal growth factor receptor 2 (HER2), epidermal growth factor receptor (EGFR), chemokine receptor 4 (CCR4), or enzymes (CD38) expressed on the plasma membranes of the cancer cells [4,8]. As noted above, mAbs can activate innate immune cells through their Fc regions and promote ADCC by NK cells and ADCP by macrophages [14,15,16,17,18,19]. Anti-SLAMF7 antibody elotuzumab also directly activates NK cells expressing this receptor in addition to inducing FcγR mediated ADCC and ADCP [20]. Some mAbs—most notably rituximab, ofatumumab, alemtuzumab, and daratumumab—also activate the complement cascade and induce CDC [2,4,8,21,22,23] (Table 1). Given the complexity of the immune activation induced by all of these mAbs, understanding which of these different mechanisms is most important for their efficacy in vivo is an important and complex question that has yet to be resolved. In particular, the role of complement in the mechanisms of action of rituximab and certain other therapeutic mAbs remains an active area of research [2]. In the next paragraphs, we will describe what is known about the role of complement in the mechanism of action of unconjugated IgG1 antibodies targeting tumor antigens, including the indirect effects that complement activation may have beyond target cell lysis.

## 2. Complement Activation by Human IgG1 mAbs

**Figure 1:**
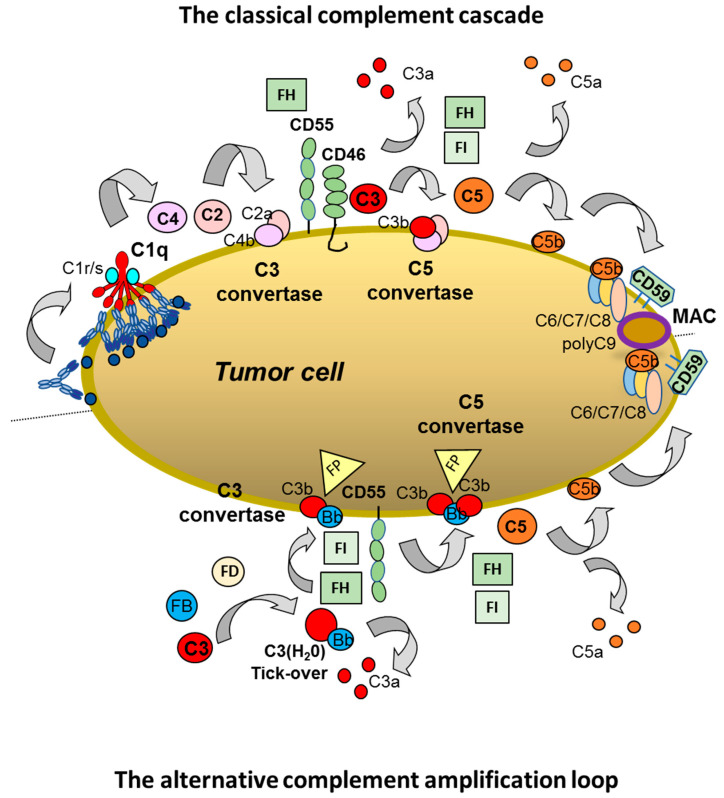
The classical and alternative complement pathways. The classical pathway (**top**): Human IgG1 antibodies bind antigen, form hexamers that allow C1q binding and the activation of the classical complement cascade. This is followed by C2 and C4 cleavage to produce the membrane bound C3 convertase (C4bC2a complex). Further cleavage of C3 to C3a and C3b forms the C5 convertase (C4bC2aC3b). C5 is cleaved to C5a and C5b allows further recruitment and activation of the C6, C7 and C8 components which catalyze C9 polymerization forming the membrane attack complex (MAC). The alternative pathway (**bottom**): it is initiated by tick-over activation of C3 in the fluid phase (C3(H_2_O)). It is further activated by Factors B and D to form the alternative C3 convertase (C3bBb) which is stabilized by Properdin (FP, yellow triangle). Further C3 cleavage forms the C5 convertase (C3bBbC3b) (also stabilized by Properdin). The alternative pathway amplifies the classical pathway. Both pathways are inhibited by the soluble inhibitors Factor H (FH) and Factor I (FI) and by membrane bound inhibitors: CD46 and CD55 at the level of the C3 convertase and CD59, which inhibits C9 polymerization. Complement pathway inhibitors are shown in green.

The main steps of the classical complement cascade on mAb-opsonized tumor cells are illustrated in Figure 1 (top). The initial binding of the IgG1 antibody to the multiple target antigens expressed on the plasma membrane is rapidly followed by aggregation and, in optimal circumstances, hexamerization of the antibody on the surface [24,25], allowing the efficient binding of the C1q (hexameric)/(C1r)_2_/(C1s)_2_ complex, which in a series of proteolytic steps sequentially leads to activation of C1r, C1s, and subsequent activation of soluble C4 and C2 to yield C4b and C2a. C4b fragments attach covalently (opsonization) to the membrane in the vicinity of the antibody or to the antibody itself, and this is followed by non-covalent binding of C2a to generate the C3 convertase enzyme (C4bC2a). The C3 convertase binds and cleaves soluble C3 into C3a, a potent anaphylatoxin, and into C3b, which binds to the C4b/C2a complex and forms the C5 convertase (C4bC2aC3b). C3b also binds covalently (opsonizes) to target acceptor sites (amino and hydroxyl groups) on the cell membrane as well as to the cell-bound IgG mAb. Cleavage of C5 by the C5 convertase produces C5a, another important anaphylatoxin, as well as C5b. Production of C5b catalyzes formation of the C5b/C6/C7/C8 complex and initiation of C9 polymerization, inducing formation of the pore-forming membrane attack complex (MAC). Insertion of a sufficient number of MACs in the membrane above a threshold level leads to the rapid lysis of the target cells [26,27,28,29,30,31].

As also described in other chapters of this series, C3 is central to the complement cascade. It is also part of the alternative pathway of complement, a pathway that relies on Factor B, Factor D, Properdin (FP), and aqueous phase hydrolyzed C3 (C3H_2_O) instead of C2 and C4, to form the C3 and C5 convertases (C3bBb and C3bBbC3b, respectively, both stabilized by Properdin) (Figure 1, bottom). C3(H_2_O) is constantly generated at low levels in a tick-over mechanism (i.e., a weak but constant hydrolytic activation of C3), but the alternative pathway can amplify complement activation (via nascent C3b) first generated by the classical pathway (Figure 1) [26,27].

In view of the cytotoxic and inflammatory nature of complement, it is not surprising that multiple independent controls serve to provide protection of normal cells and tissues from the ravages of complement. For example, C3 deposition, whether produced by the classical or alternative pathway, is tightly regulated by membrane and soluble inhibitors: C3b is rapidly inactivated (proteolyzed) to iC3b (inactive C3b) and then to C3d and C3dg and soluble C3c by Factor I (FI), a soluble protease that inhibits the complement cascade [26]. The transmembrane ubiquitous protein CD46 (membrane cofactor protein or MCP) and Factor H (FH) both act as a cofactors for Factor I and therefore enhance C3 convertase downmodulation. CD55 (decay-activating factor, or DAF) is a glycosylphosphatidylinositol (GPI)-linked membrane protein that accelerates the dissociation of C3b from the C3 convertase, thus inhibiting the cascade. Other inhibitors, in particular membrane-bound GPI-linked protein CD59, inhibit the final steps of the cascade, i.e., the polymerization of C9 for MAC formation. C3b deposition and convertase formation are central regulated steps and do not necessarily lead to MAC formation, depending on the balance between the strength of initial activation and the level of inhibition by the regulators. This is why both C3b deposition (the first phase of complement activation) and the generation of soluble C5b-9 along with MAC binding to the cells (corresponding to the last phase: MAC formation on the cell membrane) are the parameters most commonly measured to identify the first and second phase of the cascade. On this basis, it should be clear that C9 polymerization generally correlates with effective cell lysis [30,32,33].

## 3. The Interaction of Complement Components with Immune Cells

**Figure 2:**
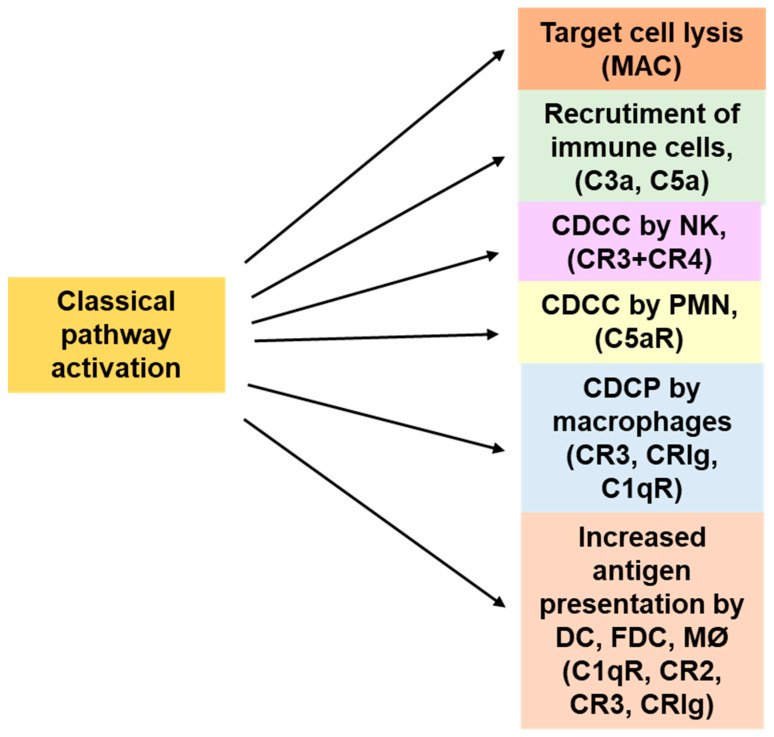
Multiple possible roles of complement for tumor control by IgG1 MAbs. Complement activation leads to complement mediated cell lysis but also to recruitment and activation of immune cells through complement fragments and their receptors which amplify the Fc-mediated ADCC and ADCP of IgG1 antibodies. CDCC: Complement dependent cellular cytotoxicity; CDCP: Complement dependent cellular phagocytosis; DC: dendritic cell; FDC: Follicular dendritic cells, MAC: membrane attack complex. MØ: macrophages; PMN: polymorphonuclear neutrophil; C1qR: C1q receptor, CR2, CR3, CR4, and CRIg: Receptors for complement fragments.

The complement cascade induced by an IgG1 mAb like rituximab may lead to the formation of the MAC and target cell lysis. However, complement is also at the center stage of a crosstalk with immune cells, and this crosstalk can be equally important to achieve immune-mediated elimination of tumor cells in vivo. For example, C3a and C5a are released following complement activation and are strong anaphylatoxins, thereby interacting with C3aR and C5aR1 (CD88) expressed on a variety of effector cells, including mast cells, macrophages, polymorphonuclear neutrophils (PMN), and dendritic cells (DCs). They can induce chemotaxis of the cells to the tumors and the generation of a profound pro-inflammatory state [26,34]. C3a and C5a also increase the permeability of small blood vessels through this inflammatory reaction and facilitate immune cell recruitment to sites of complement activation [35]. Immune cells express several different receptors for the cell-bound complement fragments—in particular, C3b and its degradation products iC3b and C3d(g)—as well as C4b and C1q. Several of these receptors, such as cC1qR (collagen C1q receptor), CR1 (complement receptor 1, CD35), CR3 (complement receptor 3, CD11b/CD18), and CRIg (complement receptor of the immunoglobulin superfamily), are implicated in the activation of macrophages and neutrophils and also function as mediators of phagocytosis and ADCC of opsonized target cells. These complex interactions are nicely reviewed by Lukacsi et al. [36]. Thus, macrophages can mediate phagocytosis of targets through both FcγRs and CRs, and the potential synergy in this process was recognized long ago [5,37,38,39,40,41]. DCs also express receptors for complement fragments such as C3a and C5a, and signaling through these receptors increase major histocompatibility complex (MHC) expression, antigen internalization, and antigen presentation. Complement factors also modulate T cell responses directly through CR1, CR2 (complement receptor 2, CD21), C1q, C3aR, and C5aRs receptors [42,43]. The multiple role of complement and complement receptors expressed by immune cells are summarized in Figure 2.

## 4. Main Factors Affecting Complement Activation by IgG1 Anti-Tumor Antibodies

### 4.1. Antigen Density and Hexamerization

The need for IgG1 hexamerization to allow for most effective chelation of hexavalent C1q and robust activation of the classical complement pathway explains why some mAbs activate complement efficiently and others do not, since the capacity to form hexamers depends on the density of the antigen on the surface, the capacity of the antibody to cluster multiple copies of the antigen, the specific orientation of the bound antibody molecules with each other, the closeness of the epitope to the cell membrane, and the specific epitope recognized [24,44,45,46,47]. For example, different anti-CD20 antibodies vary in their capacity to activate complement, with ofatumumab being the most effective, followed by rituximab [48,49] (both so-called type I anti-CD20 antibodies). Ofatumumab binds to CD20 at a site closer to the cell membrane, thus allowing for more efficient C1q binding and deposition of nascently activated C4b and C3b on the cell, and it is well-established that, for a variety of substrate cells, substantially more CDC is mediated by ofatumumab than by rituximab [48,49,50]. In contrast, obinutuzumab is only a weak complement activator [11,50] (a type II antibody). Rituximab and ofatumumab, but not obinutuzumab, are capable of relocating CD20 into lipid rafts, concentrating the antigen to small regions of the membrane, which will favor hexamerization of the antibody. In fact, the capacity of different anti-CD20 mAbs to translocate CD20 to lipid rafts correlates with their efficiency at inducing CDC [47,51]. CDC also correlates with the capacity of anti-CD20 or other antibodies to form hexamers.

Recently cryogenic electron microscopy and crystal structure studies of different anti-CD20 mAbs bound to purified CD20 or CD20 peptides have allowed for the precise analysis of the structure of type I and II anti-CD20 mAbs and their orientation with respect to CD20 itself and to adjacent antibody molecules [52,53,54]. CD20 forms a dimer rather than a tetramer as previously suggested and the studies show that each CD20 dimer binds 2 rituximab or ofatumumab Fabs (2:2 stoichiometry) but only one obinutuzumab Fab (2:1 stoichiometry), fully confirming the known binding behavior of these antibodies identified by flow cytometry. These studies altogether suggest that the epitope recognized by rituximab is more extended than previously thought and in part overlaps with that of ofatumumab. Furthermore the orientation of binding of the different mAbs to CD20 are distinct so that that rituximab and ofatumumab binding rapidly leads to CD20 concatenation and hexamerization of the mAbs on the cell surface, appropriate for C1q binding [52,53]. In contrast, obinutuzumab Fabs bind CD20 with a 2:1 stoichiometry (CD20:Fab) due to the steric hindrance between the 2 Fabs. This different orientation and steric hindrance explain its decreased ability to form hexamers and therefore to activate complement [52]. These studies nicely show how the specific epitope recognition of different mAbs can lead to different binding orientations, inter-molecular interactions, and structural constrictions that, in turn, lead to quite different capacities to activate complement, even if the mAbs are directed against the same antigen (CD20) and bind partially overlapping epitopes [47,51,54]. Thus, even though distance of the epitopes from the membrane, as well as antibody affinity, may affect CDC as well as ADCC/ADCP [55], recent data suggest that major role intermolecular interactions and the capacity of the mAbs to form hexamers are major determinants for CDC.

Similarly, among a panel of anti-CD38 antibodies, only daratumumab was found to be a potent complement activator, although the epitope recognized by daratumumab overlaps with that of other antibodies that are poor activators, suggesting that, in this case, the specific orientation of daratumumab may allow for more efficient hexamerization [21,56]. Fc mutations that favor hexamerization of antibodies targeting EGFR, CD38, or CD37 can render them strong effectors through CDC [24,25,44,45,57,58] and are being developed for clinical use [59,60].

The need for antibody clustering and hexamerization explains why the level of expression of antigen on the target cell membrane at least in part determines whether specific mAbs will be able to efficiently activate complement and lead to CDC. There is indeed a threshold level of CD20 required to allow for robust complement activation, sufficient to lead to high levels of activation of C3 followed by adequate activation of C5, thus leading to efficient downstream MAC deposition and cell lysis [4,29,61,62,63]. Indeed, CLL cells are less sensitive to CDC mediated by rituximab than most B-NHL cells that may express 10-fold higher levels of CD20 on their surfaces [63]. This rather low level of complement-mediated lysis of CLL cells in vitro by rituximab is increased considerably when using ofatumumab, in agreement with the higher capacity of the latter antibody to activate complement. Nonetheless, ofatumumab-mediated CDC is still antigen density dependent [62,64].

### 4.2. Membrane and Soluble Complement Inhibitors

CDC induced by IgG1 mAbs is also regulated by both membrane and soluble complement inhibitors that protect normal cells and tissues from complement. Cancer cells are known to express, and sometimes overexpress, the membrane complement inhibitor proteins CD46, CD55, and CD59 [33]. CD55 and CD59 have been shown to substantially inhibit the complement cascade in vitro, induced by rituximab and ofatumumab, reducing MAC binding and subsequent CDC [63,64,65,66,67,68,69,70,71,72]. Targeting the third short consensus repeat (SCR3) of CD55 with antibodies or small molecules appears to be required for CDC enhancement [73]. CD55 and CD59 activities are species-specific, explaining why the complement of some species like guinea pig are hyperactive against human cells [74]. CD55 and CD59 act synergistically to protect cells so that blocking both molecules simultaneously generally leads to the best enhancement of CDC [63,65,75]. The cooperation between CD55 and CD59 is explained by the different steps in the complement cascade that these two molecules inhibit (Figure 1).

Overexpression of CD55/CD59 also downmodulates complement-mediated lysis induced by other therapeutic IgG mAbs, such as trastuzumab in HER2-overexpressing carcinoma cell lines [76,77,78]. Blocking CD55 and in particular CD59 increased in vitro CDC of acute lymphoblastic leukemia, MM, and sarcoma cells induced by alemtuzumab, daratumumab, rituximab, and anti-CD24, respectively [79,80,81].

Whether CD55 and/or CD59 play a role in protecting cancer cells from mAb-mediated CDC in vivo is not completely clear. Increased CD55/CD59 expression was observed on cell lines selected in vitro for resistance to rituximab and complement [82]. Inhibiting CD55 and CD59 has also been shown to enhance the activity of rituximab in mouse xenograft models [83,84]. However, Williams et al. reported that CLL cells that persisted in the circulation after infusion of large amounts of rituximab had reduced levels of CD55 and CD59 due to “innocent bystander” loss of these membrane-associated proteins induced by trogocytosis of nearby CD20 [85]. As a result of the loss of CD20, these cells are resistant to rituximab-mediated complement activation, but this is clearly not due to CD55 and or CD59 up-regulation.

In contrast to CD55/CD59, there is little evidence, using antibodies, that blocking CD46 alone has any effect on IgG1 triggered CDC [86]. However, the lack of effect of anti-CD46 mAbs may be due to incomplete functional block of the protein, since an adenovirus-derived recombinant ligand of CD46, called Ad35K++, which induces cross-linking and internalization of the molecule, also significantly increased rituximab efficacy in vitro and in vivo in mouse and monkey studies [87]. These data indeed provide considerable evidence that CD46, like CD55 and CD59, modulates the efficacy of at least some IgG1 therapeutic antibodies [88].

As already mentioned above, soluble FI and FH function as inhibitors of the classical complement cascade and of the alternative pathway amplification loop by accelerating the dissociation of the C3 convertases as well as proteolytically inactivating C3b and C4b (Figure 1). FH has been shown to diminish the efficacy of ofatumumab-mediated CDC of CLL cells in vitro [89,90]. FH inhibition also enhances CDC of a subset of CLL samples and cooperates with anti-CD59 [91]. Membrane protein sialylation also inhibits complement at least in part by promoting binding of FH [92] and this has been suggested as an additional mechanism of resistance of CLL cells to anti-CD20 mediated lysis. That is, due to substantial α2-6 sialyl transferase activity, high levels of surface sialic acid are expressed on the cells, leading to binding of the complement inhibitor FH and subsequent downmodulation of complement activation [93]. FH also binds to cell surfaces and to apoptotic cells by recognition of other molecules, such as extracellular matrix proteins, DNA, soluble pattern recognition molecules, etc., thereby protecting them from complement attack [94]. With respect to FI, Lindorfer et al. reported that blocking its action increases CDC of CLL cells mediated by rituximab or ofatumumab [95]. The possible use of FI inhibition to enhance anti-tumor antibody activity is vivo is still unknown but of obvious interest.

It is clear that manipulation of the complement cascade, through the design of anti-tumor mAbs with increased ability to activate complement [44,59,96], or with mAbs that hyperactivate the complement cascade [97] or block soluble or membrane bound inhibitors [63,84,91], may all be feasible strategies to enhance the CDC activity of mAbs for cancer immunotherapy. However, there is still the need for the demonstration that each of these strategies has efficacy in vivo.

## 5. The Role of Complement in the Therapeutic Activity of Anti-Tumor mAbs

### 5.1. Studies In Vitro and in Animal Models

The importance of complement activation by unconjugated IgG1 mAbs in contributing to the anti-tumor response in vivo has been the subject of a considerable series of investigations, even for rituximab, which has been the most studied therapeutic mAb [2,4,98]. The fact that most approved IgG1 mAbs are able to activate complement upon binding to target cells suggested a positive role for complement in tumor control. Indeed rituximab [63,65], ofatumumab [48,62,70], alemtuzumab [22], daratumumab [21], trastuzumab [77], and cetuximab [99] have all been reported to activate complement in vitro, albeit with highly variable efficacity. However, other mAbs, such as isatuzumab and obinutuzumab as well as others, are effective in vivo even though they are poor complement activators [100,101,102].

Most anti-tumor IgG1 mAbs have also been demonstrated to promote ADCC and ADCP (Table 1). FcγR and cell-dependent mechanisms, ADCP in particular, have been clearly and consistently shown to be crucial for efficacy in most animal tumor models [11,103,104,105]. Murine models are, in contrast, rather discordant with regard to the role of complement in vivo for complement activating mAbs like rituximab. Some models suggest a role of complement in vivo in murine syngeneic models in which C3 knock down or complement depletion by cobra venom factor diminished or abolished the therapeutic efficacy of rituximab [2,106,107] or cetuximab [99]. However, other studies did not confirm this finding in different models [108]. Rather, most murine models suggest that FcγRs and myeloid cells are required and suggest a strong role of ADCP in the mechanism of action of many unconjugated mAbs, including rituximab, ofatumumab, obinutuzumab, cetuximab, trastuzumab, and daratumumab [11,103,108,109,110,111,112,113], as reviewed by Stevenson [114]. Some data that may reconcile some of the above-mentioned contrasting results indicate that the complement requirement for anti-CD20 activity in vivo may differ according to CD20 expression levels and tumor burden [109,115,116,117,118]. Furthermore, immune cells, including macrophages, PMN, T cells, NK cells, and dendritic cells express complement receptors, and some of these molecules participate in complement dependent cellular cytotoxicity (CDCC) or complement dependent phagocytosis (CDCP) (Figure 2) [119]. In other words, NK cells and macrophages recognize tumor-cell-associated complement fragments—in particular C3b, iC3b, and C3d and mediate cytotoxicity or phagocytosis [36]. Thus, complement may control tumor growth directly through CDC or indirectly through CDCC and CDCP as well as through the chemotaxis and activation of immune cells by C3a and C5a. These reactions may eliminate the tumor cells through either FcγRs or complement receptors or both. In support of this concept are studies of mutant anti-CD20 antibodies able to activate complement but unable to bind to FcγRs, which indicate that CDCC by NK cells and CDCP by macrophages does play a role in vitro and in vivo in a murine immunocompetent model [120]. Similarly, an immunodeficient NOD scid gamma (NSG) mouse model with active complement suggests a contribution of both complement and immune cells for tumor control by rituximab [121]. Such interactions between complement and immune cells may also explain the need for both in some in vivo models, such as the BJAB xenograft model treated with rituximab [122] and in the syngeneic EL4-CD20 model [123]. Similar cross-talk has been shown for other antibodies such as cetuximab [9] and a complement optimized anti-EGFR mAb was found to induce enhanced ADCC by PMNs [124].

Deposition of C3 fragments on B cells mediated by rituximab may have unexpected negative consequences. In a series of provocative papers, Weiner et al. reported that NK-cell-mediated killing (ADCC) of rituximab-opsonized B cells is substantially reduced if complement is activated; their results suggest that the deposited C3 fragments sterically hinder interaction of NK cell FcγRIII (CD16) with the Fc region of cell-bound rituximab. Moreover, this “problem” does not occur when obinutuzumab is examined, most likely because it poorly activates complement and C3b deposition is low and/or because it has higher affinity for FcγRIII [125,126]. Thus, complement activation may in some circumstances antagonize ADCC.

Several groups have shown that antibodies can induce a vaccinal effect; in particular, anti-CD20 mAbs induce presentation of tumor antigens (e.g., mutant proteins or aberrantly expressed differentitation antigens) to T cells by DCs [127,128]. Whereas DC antigen uptake for a vaccine effect has been shown to be mediated by FcγRs [129], complement components could also play a role, since DCs express several complement receptors [36]. However, the physiological significance of these observations is unclear; in particular, there is virtually no evidence that ofatumumab or rituximab treatment in humans promotes an immune response to either malignant or normal B cells. Furthermore the expression of complement receptors on different immune cell types including DCs is not identical between mice and men, making the study of the relevance of a possible vaccine effect via these molecules even more difficult [130].

Anti-CD38 daratumumab is an example of another mAb that eliminates tumor cells based on multiple mechanisms of action, including complement, which may synergize with each other for maximal antibody efficacy in vivo. Daratumumab, similar to rituximab, induces CDC, ADCC, and ADCP in vitro. Moreover CDC, as well as ADCC in vitro, correlates with CD38 expression levels on MM cells and is enhanced by antibodies blocking CD55 and CD59 [21,110,131]. Daratumumab and isatuximab have also been reported to deplete CD38^+^ immune regulatory cells, such as Treg, Breg, and myeloid derived suppressor cells (MDSCs) in vitro and in vivo [132,133]. We suggest that these activities may reflect, in part, daratumumab mediated “trogocytosis” of CD38 (i.e., the transfer of the target molecule, CD38, together with bound antibody from the tumor cell to phagocytes mediated by FcγRs on the acceptor cells), and so the issue may be more complex [132,134]. Finally anti-CD38 mAbs inhibit the CD38 ectoenzyme activity [135], thus diminishing immunosuppressive adenosine production. Thus the multiple effects of anti-CD38 antibodies, including daratumumab, probably all contribute to their efficacy in vivo and CDC may be dispensable in some cases [100].

### 5.2. Ex Vivo and In Vivo Human Studies

The major caveat of studies in mice is that both complement and FcγRs differ considerably between mice and humans, and some complement inhibitory proteins are species specific, so that the relative roles of CDC, ADCC, and ADCP in mice may not fully recapitulate the human situation [74,136]. For this reason, some groups have attempted to measure the role of complement and immune cells in human whole blood assays, which may at least better reflect what takes place in the circulation immediately after mAb infusion in patients [50,64,137]. The best compounds to block coagulation in these assays are hirudin and its derivatives, since these molecules block thrombin activation but do not affect the complement cascade, unlike most other anticoagulants [50,64,119]. With such assays, it was possible to show that the most effective short-term depletion of neoplastic B cells in whole blood by rituximab and ofatumumab, but not obinutuzumab, requires complement and can be blocked by anti-C5 eculizumab [50,64]. These data suggest that, at least in the circulation in humans, the first mechanism of rituximab and ofatumumab for B depletion is via CDC. These in vitro assays have limitations, since they do not fully model the flowing circulation along blood vessels and the effects of mAbs beyond 24 h of treatment.

Analyses of blood samples have been performed in patients treated with therapeutic IgG1 mAbs. Rapid complement activation with deposition of C3b and iC3b on the CLL cell membrane has been demonstrated very soon after rituximab and ofatumumab infusion [138,139]. The opsonized cells can then be effectively removed from the circulation by fixed tissue macrophages that have receptors for cell-bound IgG and C3 fragments, as first demonstrated for other substrates almost 50 years ago [37,40,41]. C5a production, as well as consumption of complement components with exhaustion of rate limiting factors, in particular C2 and C4, has been observed following rituximab or ofatumumab infusion in patients with CLL [138,139,140,141,142,143]. These data clearly show that complement is rapidly activated, and the results are consistent with the whole blood assays. Some investigators have also observed a correlation between CDC in vitro and response of CLL patients to rituximab in vivo, in support of a role of complement in vivo in humans, either directly or indirectly, by enhancing cell-mediated mechanisms [93]. Similarly, Manches et al. found a correlation between differential sensitivities of B cell lymphoma subtypes to rituximab-mediated CDC in vitro and clinical responses to rituximab. They found that lymphomas in which the patients’ primary B cells were highly sensitive to CDC (e.g., follicular lymphoma cells) showed overall a better clinical response to rituximab therapy than in neoplasias in which the B cells (CLL cells) were only weakly killed by rituximab-mediated CDC [144].

Baig et al. first reported that alemtuzumab and ofatumumab could synergize in promoting CDC of CLL B cells, reaching close to quantitative killing of the cells. CDC was not at all correlated with levels of CD55 or CD59. They then isolated B cells from the bloodstream of CLL patients soon after they were treated with ofatumumab. These cells could not be killed in vitro by CDC upon addition of more ofatumumab because CD20 had been removed from the cells due to trogocytosis. However, the sensitivity of the cells to alemtuzumab-mediated CDC remained quite high, indicating that the CD52 epitope on the cells recognized by alemtuzumab was clearly still expressed at high levels, and that complement control proteins on the cells were not upregulated to provide protection against the alemtuzumab [141,145].

The demonstrated exhaustion of complement activity and of specific complement components after antibody infusion led to the suggestion that fresh frozen plasma could be used to replenish missing factors and overcome resistance due to complement exhaustion [138]. Fresh frozen plasma has been used in CLL patients treated with rituximab with some positive results [138,146,147,148,149]. Nonetheless the efficacy of such an approach may be limited by the downmodulation of CD20 expression that follows mAb infusion and take place mostly through trogocytosis of CD20, together with bound antibody from the tumor cell to phagocytes expressing FcγRs [134,150,151,152,153,154]. Trogocytosis has been shown to occur both in vitro and in vivo in CLL patients and is likely to be another mechanism of resistance to complement and cell-mediated cytotoxicity [85]. Thus, in vivo, exhaustion of effector mechanisms, including complement and effector cells such as NK and macrophages, as well as loss of target antigen by trogocytosis or internalization, can limit the efficacy of anti-tumor mAbs [18,134,139,152,155,156].

Several analyses of polymorphic elements associated with complement related genes in relation to the clinical response of follicular lymphoma and diffuse large B cell lymphoma patients treated with rituximab have been conducted, but the results have not offered clear-cut answers about the role of complement in the efficacy of this antibody [157,158,159].

With regard to complement inhibitors, in particular CD55 and CD59, several studies have attempted to determine whether high expression levels of these proteins correlated with resistance or with relapse after mAb treatment. Although some correlations have been found in cases of lymphoma patients treated with rituximab and chemotherapy [160,161], this has not been confirmed in other studies [162], even with rituximab used as monotherapy [163]. A correlation between CD55 and CD59 expression and response of breast cancer patients to trastuzumab has been suggested [164]. Interestingly, in MM patients who progressed after daratumumab treatment, their malignant plasma cells had elevated levels of CD55 and CD59, but not of CD46, suggesting that resistance to daratumumab in vivo may be related at least in part to resistance to CDC [80]. In conclusion, the data derived from clinical correlation studies suggest that in some patients, complement may play a role in antibody therapy, but clearly, due to the multiple mechanisms of therapeutic antibodies, there are no clear-cut answers about the role of complement in vivo.

Another clue about the role of complement may come from the results of the clinical investigations with anti-CD20 mAbs and patient CLL cells, which show very different capacities to activate complement in vitro. For example, as noted above, ofatumumab is much more effective than rituximab in mediating CDC of CLL cells, and it is noteworthy that ofatumumab (but not rituximab) was approved as a single agent for the treatment of CLL [165]. On the other hand, obinuzutumab is ineffective as measured by CDC, but it is much more effective in killing CLL cells by other mechanisms and was also approved as a single agent for CLL [47].

It should thus be clear that comparison of these three mAbs is made difficult by the fact they have mechanisms of action independent of complement, and most investigations have not been performed as head-to-head comparisons. In some cases, the studies that have compared obinutuzumab with rituximab have shown a significant advantage of the former, particularly in CLL in combination with chlorambucil [166,167]. In other B-NHL types, the advantage of obinutuzumab has not been consistently demonstrated (reviewed by Pierpont [6]). In addition, the phase III clinical trials comparing obinutuzumab with rituximab have used higher doses and a different schedule of obinutuzumab, making rigorous comparison more difficult. There have also been few direct comparisons between ofatumumab and rituximab in the clinic [168]. Overall, ofatumumab seems to induce similar response rates as rituximab in B-NHL [6]. Therefore, one can conclude that the three anti-CD20 antibodies used for B-NHL and CLL treatment along with chemotherapy do not show greatly different efficacies in vivo, at least not such differences as were hoped for when they were selected and developed on the basis of their higher efficacy or different mechanisms of action in vitro [169,170]. These conclusions support the idea that the ultimate mechanisms are multifaceted, that combinations of mechanisms probably work, and some may predominate more than others in different patients, also depending upon the sites in which the tumors are targeted. Furthermore, exhaustion of most of the involved mechanisms has been shown in vivo, including complement and cell-mediated cytotoxic mechanisms [138,171], as well as down modulation of target antigen through trogocytosis [134]. This means that an antibody with a greater CDC or ADCC potential in vitro may in any case be limited in vivo by these mechanisms [2,141,172].

## 6. Conclusions and Future Perspective

Unconjugated IgG1 anti-tumor mAbs often show a variety of mechanisms of action that may operate simultaneously and interact with each other: neutralization of the target antigen/receptor, activation of cell-mediated cytotoxicity, and complement activation. More recent evidence suggests that these mechanisms probably interact with each other, either positively or negatively. In particular, evidence suggests that complement factors and receptors synergize to enhance ADCC and ADCP. Complement fragments may also affect T-cell-mediated immunity by interaction with DCs and T cells, perhaps explaining the delayed or long-term effects of antibody treatment that has been suggested in some cases [173]. Also, the direct effects of the mAbs can, in some cases, synergize with immune-mediated mechanisms, as is the case for NK activation by anti-SLAMF7 mAb elotuzumab [174] or the T-cell-activating effect of anti-CD38 mAbs [100]. Given this plethora of activities and interactions, understanding the relative contribution of each of the potential mechanisms of action of IgG1 therapeutic mAbs is difficult to clearly establish and remains unsolved even for the best-known mAbs. In particular, a substantial literature of in vitro studies and in vivo correlations cited in this review strongly support (but do not prove) the importance of complement in the action of several anti-tumor mAbs. These observations and the finding that exhaustion mechanisms may limit efficacy should, in our view, lead to development of optimized schedules/combination treatments in the clinic, including combination with cell therapy approaches, as well as optimized mAbs capable of multiple effector functions. The latter is becoming possible thanks to a better understanding of mutations or modifications that may enhance different mechanisms (CDC, ADCC, ADCP) and perhaps abolish the negative interference that is sometimes observed between these effects or through the use of mAb combinations that may be specifically favorable for these mechanisms [175]. The effects of chemotherapy on the different mechanisms of unconjugated therapeutic IgG1 antibodies is another area of particular interest, and it will be important to identify the best drug combinations and schedules required to achieve synergy between small drug and mAb therapy. Indeed, chemotherapy may negatively affect the cell-mediated mechanisms of therapeutic mAbs, but could also potentially affect complement mediated mechanisms, if modulation of target antigen or of complement factors is induced by drug treatment [176,177]. A precise understanding of these interactions will therefore be needed for optimized treatments.

## Figures and Tables

**Table 1:** Approved unconjugated IgG1 monoclonal antibodies (mAbs) targeting tumor antigen.

Name	Target Antigen	Antibody Type	1st Indication	Year of 1st Approval ^1^	Major Mechanism of Action
Rituximab	CD20	Chimeric IgG1	B-NHL	1997	CDC, ADCC, ADCP
Ofatumumab	CD20	Human IgG1	CLL	2009	CDC, ADCC, ADCP
Obinutuzumab	CD20	Humaniz. IgG1, Glycoengin.	CLL	2013	ADCC, ADCP, PCD
Daratumumab	CD38	Human IgG1	MM	2015	CDC, ADCC, ADCP, neutral.
Isatuximab	CD38	Chimeric IgG1k	MM	2020	Neutral. ADCC, ADCP
Alemtuzumab	CD52	Humanized IgG1	CLL	2001	CDC, ADCC, ADCP
Elotuzumab	SLAMF7	Humanized IgG1	MM	2015	ADCC. NK agonist, ADCP
Mogamulizumab	CCR4	Humanized IgG1, low fucose	T leuk/lymph	2012 Japan 2018 EU	ADCC, ADCP, Treg elimin.
Trastuzumab	HER2	Humanized IgG1	Breast cancer	1998	ADCC, neutral.
Pertuzumab	HER2	Humanized IgG1	Breast cancer	2012	Neutral. (HER2/HER3 dimerization)
Cetuximab	EGFR	Chimeric IgG1	CRC	2004	Neutral., ADCC, CDC
Panitumumab	EGFR	Human IgG2	CRC	2006	Neutral., PMN mediated ADCC
Necitumumab	EGFR	Human IgG1	NSCLC	2015	ADCC, neutral.
Dinutuximab	GD2	Chimeric IgG1	Neuroblastoma	2015	CDC, ADCC, ADCP

^1^ Food and Drug Administration (FDA) and/or European Medicines Agency (EMA) approval. B-NHL: B- Non Hodgkin’s lymphoma; CLL: Chronic lymphocytic leukemia; MM: Multiple myeloma; CRC: Colorectal carcinoma; NSCLC: Non small cell lung carcinoma; CDC: Complement dependent cytotoxicity; ADCC: Antibody dependent cellular cytotoxicity; ADCP: Antibody dependent cellular phagocytosis.
